# How the nations should gear up for future pandemics?

**DOI:** 10.7189/jogh.11.03075

**Published:** 2021-05-15

**Authors:** Alwin Issac, VR Vijay, Nadiya Krishnan, Jaison Jacob, Shine Stephen, Rakesh Vadakkethil Radhakrishnan, Manju Dhandapani

**Affiliations:** 1 College of Nursing, All India Institute of Medical Sciences (AIIMS), Bhubaneswar, India; 2 National Institute of Nursing Education, Post Graduate Institute of Medical Education and Research (PGIMER), Chandigarh, India

Health authorities from the city of Wuhan, China, informed World Health Organization (WHO) about an increase in pneumonia cases of unknown origin on December 31, 2019. Health authorities in China detected novel coronavirus as the causative agent for the pneumonia cases and the virus was initially named “2019-nCoV”, which was later renamed as coronavirus disease 2019 (COVID-19). Owing to the virus virulence and its contagious nature, WHO declared novel coronavirus outbreak a public health emergency of international concern. With the steep rise in number of people infected with the virus outside China, WHO stated the eruption as a pandemic on March 11, 2020 [1].

Owing to the plight of wildlife habitat, poor health system, and global connectivity, COVID-19 wouldn’t be the last time a virus would jump from animal into humans and endanger humanity. The ever-rising trend of globalization and human migration for trade, commerce, and tourism across the national borders has made it easy for an outbreak to progress to a pandemic. A recent study reiterates that regions in Africa and parts of Asia are more vulnerable for zoonotic diseases [2]. A spillover of infection from animals to humans might go unnoticed without a robust surveillance system and access to health care for all.

This article examines the strategies that stood apart in curbing the COVID-19 among certain countries in terms of their health care system, technology, communication, evidence based practice, and plan of action.

− Universal Health Coverage (UHC) assisted nations which had them well placed, in quickly launching their anti-epidemic action plans at the grassroots level by expanding primary health care systems.
− Surveillance, testing, contact tracking, and vigilant quarantine were all made easier, thanks to advanced technologies.
− The most important tool in risk management was proactive communication and knowledge sharing, which enabled the public to adopt protective behavior, enhanced disease monitoring, reduced uncertainty, and promoted the efficient use of resources, all of which were necessary for an effective response.
− EBP, in conjunction with cutting-edge technology, played a critical role in containing the pandemic by establishing protocols for monitoring, diagnosis, treatment, and vaccine production to ensure optimum cost-effectiveness for the public.
− Nations must prepare for a pandemic by planning, implementing, re-designing, and translating evidence into national and sub-national levels, and these plans should be updated on a regular basis to reflect changes in global guidelines, scientific evidence, and historical experiences in order to prevent future pandemics.

## UNIVERSAL HEALTH COVERAGE

COVID-19 pandemic has stretched the health care system around the world, testing their capability to cater to patient needs and protecting their health care workers. While some countries could execute their pandemic-response plans swiftly, others failed to act quickly and their health care systems have been overwhelmed [3]. Outbreak mitigation relies on expeditious diagnosing and isolation of cases; wherein, less severe cases are quarantined at home and severe cases are managed at hospital. Universal Health Coverage (UHC) or Medicare for all is incentivized to vest in cost-efficient prophylactic services, which can ward off grievous clinical outcomes and pricey treatment. Obstacles to timely diagnosis and case isolation not only affect on an individual basis, but bring forth wide societal risk [3]. Robust financing structure and investment in quality primary health care is the key to attain UHC in any country. Although government funded health care system provides access to health care for all individuals; in an emergency, attributing to their lengthy wait times and over usage, it may fail to meet the need as was seen in England [3]. Escalated out of pocket expenditure in private health care system could detrimentally devoid the financially poor in procuring the health care facilities. A government monitored and regulated public-private partnered health care system could tackle crisis effectively as was seen in Canada, South Korea, and Taiwan [3]. In any emergency, primary care remain as the initial point of contact between the public and the health system, and countries with a strong primary health care (PHC) could prevent outbreaks, protect the public from further spread of infection, prevent overburdening in hospitals, and safeguard their health care workforce [3,4]. During the Ebola virus disease outbreak in West-Africa, PHC was phenomenal in building community and country resilience. PHCs are critical to accomplish global health security and back up resilient health systems as a foundation for UHC. Investment in PHC workforce, good governance, well-functioning health-information system, sound system of procurement and supply of medicine, and health technology are critical elements to ensure access to essential health care [4]. UHC can be provided through a single payer system (Taiwan) or in public-private partnership (South-Korea) [3]. A single payer system would amalgamate administrative expenses, reduce overhead, authorize pharmaceutical fare negotiation, curtail executive pay, accelerate case reporting, and alleviate cost barrier [5]. Countries that could combat COVID-19 effectively had attained UHC with their relentless investment in health care system, namely New-Zealand, Japan, South-Korea, Thailand, Canada.

## DIGITAL TECHNOLOGY

Innovative technologies have assisted in prompt recognition of coronavirus and keep updated the data globally. Countries that reported low mortality rate from COVID have common strategies, namely surveillance, testing, contact tracing, and vigilant quarantine. Co-ordination and data management mandated for efficacious execution of these schemes relied on digital technology in collaboration with artificial intelligence. Global positioning systems, quick response codes, card transaction logs, and closed-circuit televisions were made use of to fortify contact tracing [6]. Drones, autonomous robots, self-driving cars, and facial recognition technologies were used to ensure contact less movement and deliveries [6]. Thermal scanner, biometric fever screening, and drive-through screening centers were installed for safe and effective screening to forestall a massive community outbreak [3,6]. Smart-watch and mobile applications were developed, which warned people when they came within specified distance from places where confirmed cases had been [3]. Technology can help forbid the outspread, educate the public, notify danger, endowing those on the ground to be aware of the situation, thus lessening the impact. Governments, who possessed robust and resilient technologies had real-time situational awareness, which enabled them to build more responsive strategies to prevent further damages [6]. While the viruses coalesce of deadliness and contagiousness challenged every nation, Artificial Intelligence (AI) based “chatbot” technology, telemedicine, data platforms, and contact tracing tools were instrumental in curtailing the spread of pandemic in South Korea, Germany, Australia, Thailand, and China [3]. The post-COVID era would seek for a new social contract, and technology would play an ever-important role in society.

**Figure:**
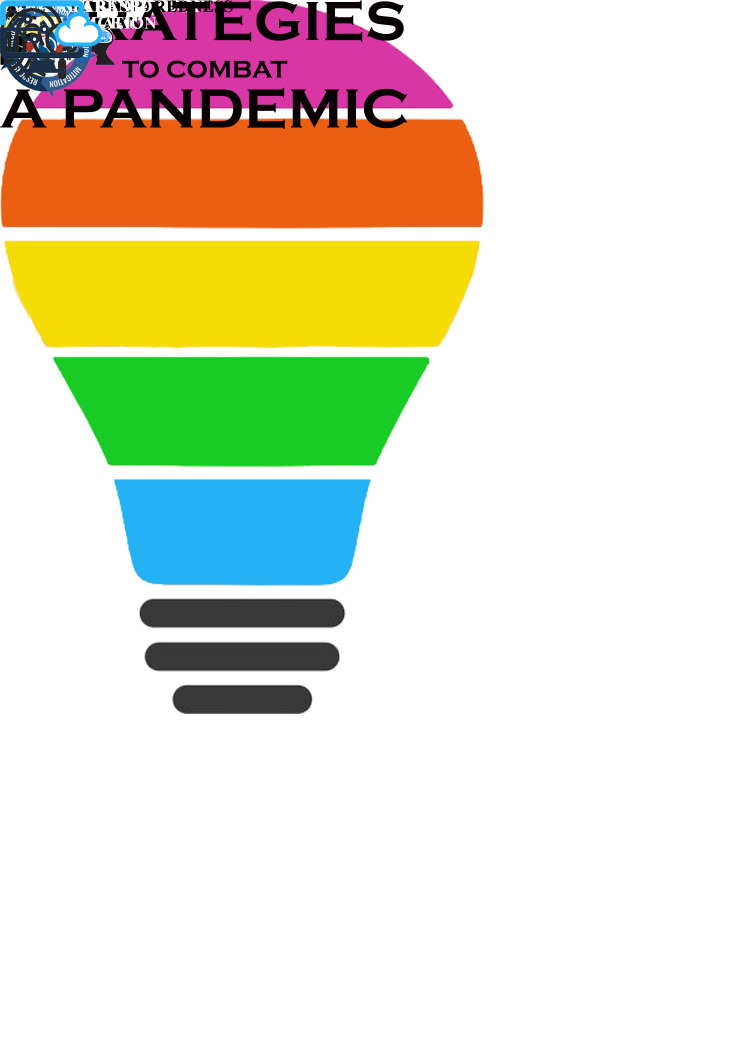
Photo: Strategies to combat pandemic (designed by the authors, used with permission).

## TRANSPARENT AND PRO-ACTIVE COMMUNICATION

Numerous instances of misinformation and disinformation have surfaced that has grossly regressed many nation’s response to the pandemic by undermining trust, amplifying fears, and sometimes leading to harmful behaviors [7]. A transparent communication about ‘what is not known’ is as important as communication about ‘what is known’. Ensuring public faith necessitate an open bipartisan communication. In nations where people’s faith on their government is deficient, attempts are to be made to build reliance before an emergency occurs. In times of public health emergency, treatment options would be scarce, organizing direct interventions would mandate time, and resources would be scanty [7]. Pro-active communication would be the most important tool in managing risk, which would allow public to embrace protective conduct, alleviate enhanced disease surveillance, bring down confusion and promote effective use of resources, all of which are requisite for an efficacious response[8]. Investment in digital technology and attaining universal health coverage may not be feasible for low and middle-income countries; while, evidences suggest that usage of face mask, maintaining social distancing, and hand hygiene practices were efficient norms in halting the spread of current pandemic [9]. However, all these required public support and it wouldn’t be possible without gaining public trust in the government, which in turn is possible through transparent communication of the facts. The widespread use of social media, namely Facebook, WhatsApp, and Instagram played a pivotal role in swiftly disseminating and empowering the public with the right information regarding managing a pandemic. South Korea, Ghana, and Germany reiterated the necessity and efficacy of proactive communication in halting a pandemic [7,8].

## EVIDENCE BASED PRACTICE

Evidence based practice (EBP) is painstaking, explicit, and sensible usage of present best evidence in making determination about care of each individual patient. The foundation of EBP is laid on the integration of most relevant evidence, physician’s clinical expertise, and the patient preferred values and preferences [10]. The pace with which the pandemic spreads is a menace to traditional models of knowledge translation and practice change. There were instances of public outcry in United Kingdom and United States of America owing to their policies that lacked evidence in COVID-19 management [3]. While in Germany, their chancellor being a research scientist formerly, paved way for their early and diligent evidence based response to COVID-19 [3]. The policy decision makers, health authorities, and clinicians need to be nimble in their reasoning and practice in order to find the right time to change. The adoption of novel methods should be based on clinical judging, weight of evidence and the equipoise of chances that any novel technique, test, or treatment might work.

## PANDEMIC PREPAREDNESS PLAN

Pandemics were once predicted to be a ‘once in century event’. But with extent of social and environmental changes driven by humans, it wouldn’t be a surprise to witness another pandemic in a few years [2]. Preparing for pandemic is an unrelenting procedure of designing, executing, re-designing, and transforming it into national and sub-national preparedness and response plans. It should be periodically revised at par with changes in worldwide guideline or in accordance to scientific base, past experiences, and or alteration in national or international statute law associated to communicable disease prevention and control [11]. While preparing a pandemic preparedness plan, pandemics of varying severity should be considered; though the response should be rooted on the existent state of affairs determined by national and global risk appraisals. During times of public health emergency, focus should be on to strengthening existing systems in place; while those novel pandemic preparedness plans must be tried out during the inter-pandemic periods [11]. In one of its kind, ‘Exercise Cygnus-2016’, a 3day simulation exercise was carried out by National Health Service, England to estimate the impact of a hypothetical H2N2 influenza pandemic on United Kingdom. The purpose of such a simulation was not only to test how emergency plans would hold up under strain, but also to acclimatize the health authorities to the sort of decisions they would be forced to make [12]. Countries that had pandemic preparedness plan in place could mitigate risk, minimize the impact, and swiftly resume activities [3,4]. It is also imperative to have the preparedness plan tailored in accordance to the socio-economic and health compliance behavior of a nation, with a periodic revision in their plan of action. There should be adequate research financial support for health care and public health research, so as to get equipped with necessary amenities to combat future pandemics [10].

## CONCLUSION

Preparation for any pandemic requires relentless and laborious effort from all the sectors of the society. Those countries that set a benchmark in COVID-19 management, reiterates their inter-sectoral coordinated effort. This pandemic has reiterated a ubiquitous truth that each individual is only as safe as the tenderest individual in the community. Universal health coverage, digital technology, transparent and proactive communication, evidence-based practice, and pandemic preparedness plan wouldn’t have stopped the pandemic from its occurrence; but it has certainly dented the intensity of affliction in nations that practiced it.
